# Development of a drug–device combination for fluorescence-guided surgery in neuroendocrine tumors

**DOI:** 10.1117/1.JBO.25.12.126002

**Published:** 2020-12-09

**Authors:** Servando Hernandez Vargas, Christie Lin, Julie Voss, Sukhen C. Ghosh, Daniel M. Halperin, Solmaz AghaAmiri, Hop S. Tran Cao, Naruhiko Ikoma, Adam J. Uselmann, Ali Azhdarinia

**Affiliations:** a The University of Texas Health Science Center at Houston, The Brown Foundation Institute of Molecular Medicine, McGovern Medical School, Houston, Texas, United States; b OnLume, Inc., Madison, Wisconsin, United States; c The University of Texas MD Anderson Cancer Center, Department of Gastrointestinal Medical Oncology, Division of Cancer Medicine, Houston, Texas, United States; d The University of Texas MD Anderson Cancer Center, Department of Surgical Oncology, Division of Surgery, Houston, Texas, United States

**Keywords:** intraoperative imaging, fluorescence-guided surgery, fluorescence system instrumentation, cancer-targeted agent, somatostatin receptor, dual labeling

## Abstract

**Significance:** The use of cancer-targeted contrast agents in fluorescence-guided surgery (FGS) has the potential to improve intraoperative visualization of tumors and surgical margins. However, evaluation of their translational potential is challenging.

**Aim:** We examined the utility of a somatostatin receptor subtype-2 (SSTR2)-targeted fluorescent agent in combination with a benchtop near-infrared fluorescence (NIRF) imaging system to visualize mouse xenografts under conditions that simulate the clinical FGS workflow for open surgical procedures.

**Approach:** The dual-labeled somatostatin analog, Ga67-MMC(IR800)-TOC, was injected into mice (n=24) implanted with SSTR2-expressing tumors and imaged with the customized OnLume NIRF imaging system (Madison, Wisconsin). *In vivo* and *ex vivo* imaging were performed under ambient light. The optimal dose (0.2, 0.5, and 2 nmol) and imaging time point (3, 24, 48, and 72 h) were determined using contrast-to-noise ratio (CNR) as the image quality parameter. Video captures of tumor resections were obtained to provide an FGS readout that is representative of clinical utility. Finally, a log-transformed linear regression model was fitted to assess congruence between fluorescence readouts and the underlying drug distribution.

**Results:** The drug–device combination provided high *in vivo* and *ex vivo* contrast (CNRs>3, except lung at 3 h) at all time points with the optimal dose of 2 nmol. The optimal imaging time point was 24-h post-injection, where CNRs>6.5 were achieved in tissues of interest (i.e., pancreas, small intestine, stomach, and lung). Intraoperative FGS showed excellent utility for examination of the tumor cavity pre- and post-resection. The relationship between fluorescence readouts and gamma counts was linear and strongly correlated (n=334, $R^{2}=0.71$; r=0.84; P<0.0001).

**Conclusion:** The innovative OnLume NIRF imaging system enhanced the evaluation of Ga67-MMC(IR800)-TOC in tumor models. These components comprise a promising drug–device combination for FGS in patients with SSTR2-expressing tumors.

## 1 Introduction

Surgery is an essential treatment option for most solid tumors and can be curative if complete resections are achieved.^1^ To improve intraoperative detection of tumors and potentially improve surgical outcomes in cancer patients, surgeons increasingly use fluorescence-guided surgery (FGS) to augment feedback obtained through standard visual and tactile cues.^2^ FGS relies on the combined administration of a fluorescent contrast agent (i.e., drug) and its subsequent detection with an imaging system (i.e., device); however, translational research strategies may consist of drug only, device only, or drug–device combinations.^3^^,^^4^

FGS has traditionally been used for detection of tissue perfusion using nonspecific contrast agents injected intravascularly, with their diffusion being merely a function of vascular flow dynamics. The metabolic clearance of the dye then provides a secondary application based on the clearance mechanism specific for the dye: indocyanine green (ICG) undergoes hepatic clearance and thus can illuminate the biliary tree; methylene blue undergoes renal clearance and can be used to identify the ureters.^5^ In addition to use for intraoperative applications, such as lymphatic mapping and sentinel lymph node biopsy, ICG has been used to assess the clinical performance of newly developed imaging systems that seek regulatory approval for human use.^6^ The current challenge in tumor localization with FGS is the lack of tumor selectivity of ICG^7^ and subsequent inability to provide adequate tumor-to-background ratios (TBRs), which limits the utility of this modality for intraoperative decision-making.^8^ The introduction of tumor-targeted FGS drugs into clinical trials has shown that cancer-specific agents may improve the predictive value of the fluorescent signal and could play an important role in cancer treatments due to their potential to enhance complete resection rates and patient outcomes.^9^^–^^14^ However, approaches that simultaneously develop tumor-specific drugs and sensitive imaging devices are needed to assess the accuracy of FGS for tumor localization and determine the translational potential of emerging technologies.

Gastroenteropancreatic neuroendocrine tumors (GEP-NETs) are generally indolent neoplasms that arise in the pancreas and gastrointestinal tract, with a propensity for nodal and liver metastases, which are found in 40% to 70% of patients at the time of diagnosis.^15^ While surgery is the primary treatment option for localized tumors and can be curative, it is also commonly employed in metastatic GEP-NETs to minimize symptoms of hormonal hypersecretion^16^ and has been associated with improved survival.^17^ Surgical outcomes in GEP-NETs are critically dependent on localizing tumors intraoperatively but are complicated by their small size (<1  cm) and multifocal presentation, which can lead to high rates of incomplete resection. ^18^^–^^22^ Conversely, reliance on palpation alone to guide surgery for multifocal GEP-NETs may result in excessive resection of uninvolved segments. Given the limited benefit of non-targeted dyes in this patient population,^23^^–^^25^ we previously developed a fluorescent agent that specifically targets somatostatin receptor-subtype-2 (SSTR2), a cell-surface receptor that is overexpressed on the majority of NETs.^26^ SSTR2-targeting strategies have displayed exceptional diagnostic accuracy and have a long history of use in nuclear medicine for detecting, staging, and treating NETs.^27^^–^^29^ Although somatostatin analogs have undergone iterative optimization over the years, the SSTR2-targeting moiety has remained relatively constant and is a validated pharmacophore for the development of targeted agents.^30^ Accordingly, we converted the clinically approved radiopharmaceutical, Ga68-DOTA-TOC, into a fluorescent counterpart, Ga68/67-MMC(IR800)-TOC, that was dual-labeled with radioactive gallium (Ga68: $t_{1/2}=68\;\min$ or Ga67: $t_{1/2}=3.3\;d$) to enable quantitative assessment of agent performance. Consistent SSTR2 specificity was observed at the multiscale level, which included cancer cells, xenografts, and biospecimens obtained from patients with pancreatic NETs.^31^ Direct comparison of Ga68/67-MMC(IR800)-TOC to Ga68-DOTA-TOC was critical to benchmark the imaging properties of our agent against a clinical gold standard and indicated excellent potential for translational FGS studies.^26^

Preclinical evaluation of tumor-specific FGS drugs is commonly performed using imagers optimized for conditions that do not recapitulate factors found in the operating room.^6^ Disparity in optical specifications, performance, and imaging conditions between preclinical and clinical imaging devices further hinders assessment of the translational feasibility of novel agents. Major differences include (i) lower sensitivity that limits microdose (i.e., sub-pharmacologic) detection, which has significant translational implications,^3^^,^^32^ (ii) long exposure times that eliminate real-time functionality, and (iii) control of known environmental factors that degrade image quality. An example of (iii) is that, unlike the controlled research environment, fluorescence imaging in the operating room is complicated by harsh lighting from potential sources that contribute to background ambient lights, such as sunlight streaming through windows, overhead lights (various bulb types), computer monitors [e.g., liquid crystal display (LCD) and light-emitting diode (LED)], surgeon headlights (e.g., Xenon and LED), and surgeon’s visualization equipment (e.g., endoscope or microscopes with Xenon or LED). Assessing the performance of novel FGS drugs with a clinical imaging system could address this limitation. However, clinical FGS imagers, whether for open or minimally invasive surgery, are designed for the spectral properties of ICG^33^ and would require hardware modifications to provide similar customized detection of a new FGS drug with differing spectral properties. Therefore, we evaluated the feasibility of using Ga67-MMC(IR800)-TOC with a benchtop clinical prototype imager (OnLume, Inc., Madison, Wisconsin) that was optimized for sensitivity to detect low doses of the drug. The drug–device combination showed excellent utility for *in situ* visualization of pre-resection fluorescence and post-resection residual fluorescence under ambient light.^34^

**Fig. 1:**
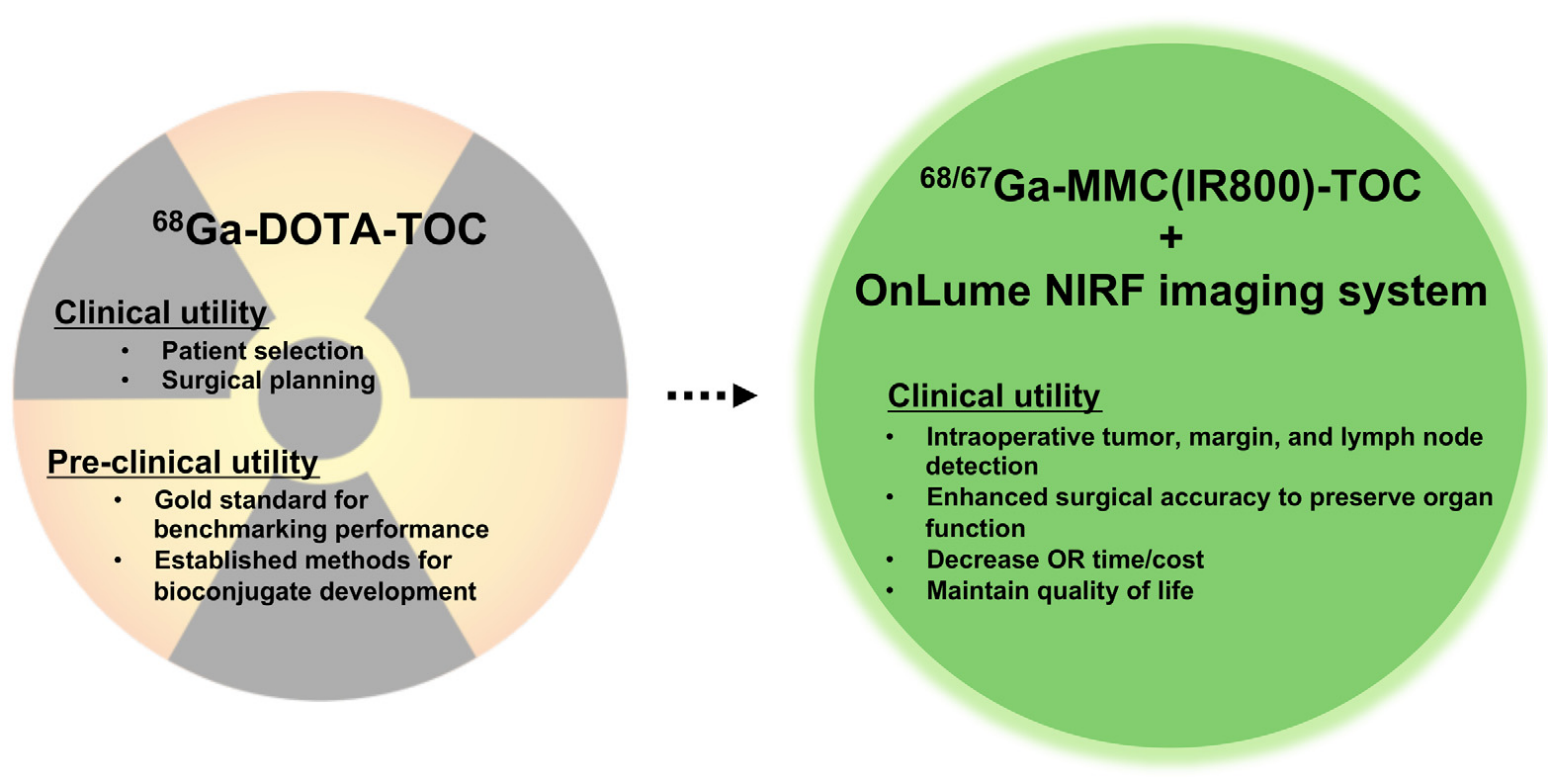
Translating the visual benefits of targeted preoperative NET imaging agents into the operating room.

Here, we characterized the drug–device combination consisting of Ga67-MMC(IR800)-TOC and the OnLume near-infrared fluorescence (NIRF) imaging system (Fig. 1) under conditions that simulate the clinical FGS workflow for open surgical procedures, such as real-time fluorescence visualization. Two key clinical endpoints for an FGS drug, namely, the optimal injection dose and imaging time point, were determined in SSTR2-expressing xenograft models using contrast-to-noise ratio (CNR) as the image quality parameter. We also describe for the first time how the radioactive properties of a dual-labeled FGS drug can be used in combination with fluorescence readouts to correlate signal acquisition by the device with the underlying drug distribution in tissues.

## 2 Materials and Methods

### 2.1 General Methods

All chemicals were purchased from Sigma-Aldrich unless otherwise noted. Reversed-phase high-performance liquid chromatography (HPLC) was performed on an analytical Hitachi LaChrom system using a Kinetex C18 column (2.6  μm) (Phenomenex) with a mobile phase of A=0.1% TFA in $H_{2}O$ and B=0.1% TFA in ${\mathrm{CH}}_{3}\mathrm{CN}$ (gradient: 0 min, 10% B; 12 min, 90% B); flow rate, 1  ml/min. Radiochemical purities of ≥95% were assessed by radio-HPLC using a dual scan-RAM (LabLogic).

### 2.2 ^67^Ga labeling of MMC(IR800)-TOC

MMC(IR800)-TOC was radiolabeled with Ga67 ($t_{1/2}=3.3\;d$) using cation exchange chromatography and purified as previously described.^31^

### 2.3 Animal Model

Athymic female nu/nu mice (Charles River Laboratories) were housed under standards of the Institutional Animal Care and Use Committee of the University of Texas Health Science Center at Houston and maintained on alfalfa-free rodent chow. SSTR2 overexpressing HCT116 (HCT116-SSTR2) cells were cultured as previously described.^31^ For all procedures, mice were anesthetized with 1% to 2% isoflurane. For xenografting, 6 to 8 week old mice were subcutaneously injected with $5\times 10^{6}$ HCT116-SSTR2 cells in Matrigel (Corning):PBS (1:1) in the shoulder (n=24). Studies were conducted 2 to 3 weeks post implantation when tumor size reached ∼5- to 10-mm maximum diameter. Overdose of anesthesia followed by cervical dislocation was the method of euthanasia for mice in terminal studies.

### 2.4 FGS Device Optimization for MMC(IR800)-TOC Imaging

The benchtop NIRF imaging system (OnLume, Inc.) is an imaging device customized for NIRF imaging of MMC(IR800)-TOC [peaks of Excitation/Emission (in PBS): 778/795  nm] with minimal background noise. Onboard the imaging systems are white light (WL) and NIRF sources that provide homogeneous illumination, and WL reflectance and fluorescence imagers that acquire images simultaneously. The wide-field imaging device can provide fluorescence overlay on WL reflectance images at video rate in real-time with no measurable change in image quality in presence of ambient light. Three emission filter sets were tested on *in vitro* samples to measure emitted fluorescent wavelengths that reach the imager to determine optimal image contrast and sensitivity. To achieve this, two-fold serial dilutions of MMC(IR800)-TOC in deionized water were prepared *in vitro* (molarity range: 3 to $1\times 10^{5}\;\mathrm{nM}$) and images were acquired at a working distance of 39 cm between the imager and the surface of the ROI. CNRs for each filter set were characterized and compared. CNRs were calculated as described in the section titled “Image analysis and image quality parameters.”

### 2.5 *In vivo*, *ex vivo* NIRF Imaging and Biodistribution Studies for Dose and Time Point Selection

For the dose-finding study, mice with HCT116-SSTR2 xenografts were intravenously injected with 2 (5.7  μg, 20  μCi), 0.5 (1.4  μg, 5  μCi) and 0.2 (0.6  μg, 2  μCi) nmol of Ga67-MMC(IR800)-TOC and imaged 24-h post-injection. Studies for identification of the optimal imaging time point were conducted with the optimal dose at 3, 24, 48, and 72 h post-injection.

Longitudinal *in vivo* and *ex vivo* NIRF images of selected tissues were acquired using both the benchtop imaging system (OnLume) and the *In-Vivo* Xtreme (Bruker) preclinical small animal imaging device (excitation and emission set to 760 and 830 nm, respectively). Image acquisition parameters for each imaging device remained consistent throughout the study.

We performed imaging with the OnLume benchtop system in an animal procedure room with sunlight passing through windows, overhead fluorescent tube lights, and one computer monitor (LCD). Sources of ambient light were not modified (i.e., monitor and overhead room lights were not dimmed and the window was not screened).

At the completion of the imaging studies, tissues were weighed and counted for radioactivity using a Cobra II auto-γ counter (Packard) that was set to correct for radioactive decay, thereby normalizing measured gamma counts to the time of fluorescence imaging. The total injected activity per mouse was determined from an aliquot of the injected solutions. The results are expressed as a % of the injected dose per gram of tissue (%ID/g) and represent the mean±SD of n=4  mice/dose or time.

### 2.6 Immunohistopathology

Suspected residual tumor tissues identified during intraoperative FGS were cryo-conserved, sectioned, and stained with hematoxylin & eosin (H&E) and immunohistochemistry as previously described.^31^

### 2.7 Image Analysis and Image Quality Parameters

Image analysis was performed with the proprietary software accompanying each imaging system. For *in vivo* TBRs and CNRs, tumor fluorescence was measured with respect to fluorescence in the hind leg as a surrogate for adjacent normal tissue. *Ex vivo* TBRs and CNRs were measured with respect to selected tissues of interest (i.e., NET-associated organs).

TBR ratio was calculated as

$$\mathrm{TBR}=S_{t}/S_{b}.$$ (1)

For fluorescent signal in the tumor $S_{t}$ and in background tissue $S_{b}$.

CNR was calculated as

$$\mathrm{CNR}=(S_{t}-S_{b})/\sigma_{b}.$$ (2)

For the standard deviation (SD) of fluorescent signal in background tissue $\sigma_{b}$.

### 2.8 Statistical Analysis and Curve Fitting

Statistical analysis and log-transformed linear regression model fitting were performed with GraphPad Prism 8.1.0. Group comparisons (n=2) were performed with two-tailed Mann–Whitney tests. Multiple comparisons (n>2) were performed with one- or two-way ANOVA (Holm–Sidak correction). Family-wise significance and confidence levels were set to P<0.05. All data are presented as mean±SD.

## 3 Results

### 3.1 Device Optimization for MMC(IR800)-TOC

**Fig. 2:**
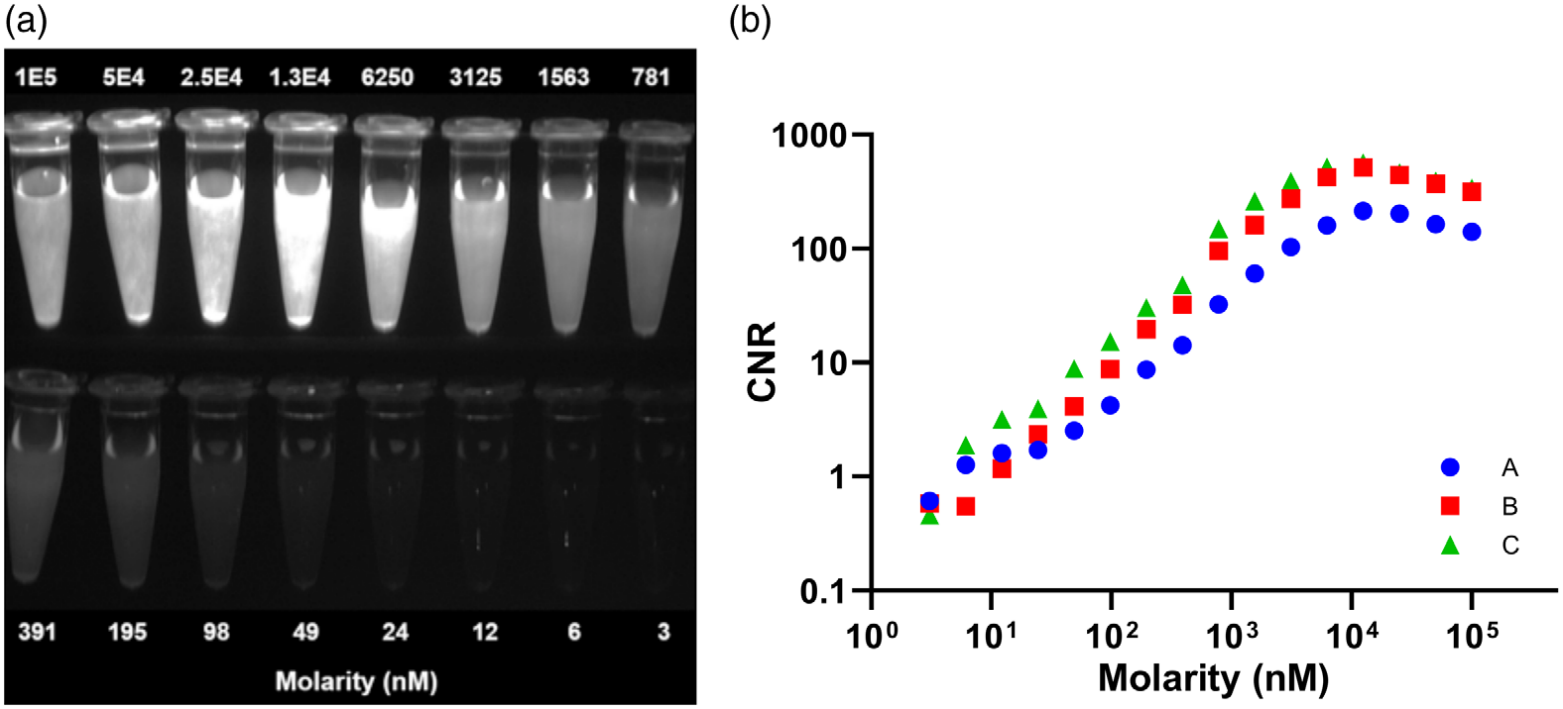
*In vitro* imaging of MMC(IR800)-TOC with the OnLume NIRF imager for contrast optimization. (a) Fluorescence-only image of two-fold MMC(IR800)-TOC serial dilutions for filter Set C. (b) CNR of MMC(IR800)-TOC dilutions measured with three different emission filter sets. Reproduced with permission from Ref. 34.

The OnLume imager was customized for optimal MMC(IR800)-TOC imaging under ambient light. The complete *in vitro* dilution series of MMC(IR800)-TOC (range: 3 to $1\times 10^{5}\;\mathrm{nM}$) was imaged in a single field-of-view [Fig. 2(a)]. For all optical filter sets, we qualitatively observed a concentration-dependent increase in fluorescence that peaked at $1.3\times 10^{4}\;\mathrm{nM}$, followed by a decrease in fluorescence at concentrations $\geq 2.5\times 10^{4}\;\mathrm{nM}$. The reduction in fluorescence at high MMC(IR800)-TOC concentrations is presumably due to dye-conjugate aggregation and quenching, which are known phenomena associated with NIRF dye conjugates.^35^ Plotting CNR as a function of drug concentration was in accordance with the qualitative assessment for all optical filters [Fig. 2(b)]. We found that while filter A yields a similar function compared to filters B and C, the provided CNR largely underperforms. On the other hand, filters B and C had similar CNRs. However, filter C outperformed filter B with a 1.6±0.7-fold higher CNR on average across all concentrations. Remarkably, in concentrations $<1.5\times 10^{3}\;\mathrm{nM}$, we gained a 1.9±0.7-fold higher CNR on average using filter C as opposed to filter B. Thus, filter C was selected for all subsequent imaging studies due to its consistently higher CNR.

### 3.2 *In vivo* Imaging and Tumor Delineation in Clinically Relevant Conditions

**Fig. 3:**
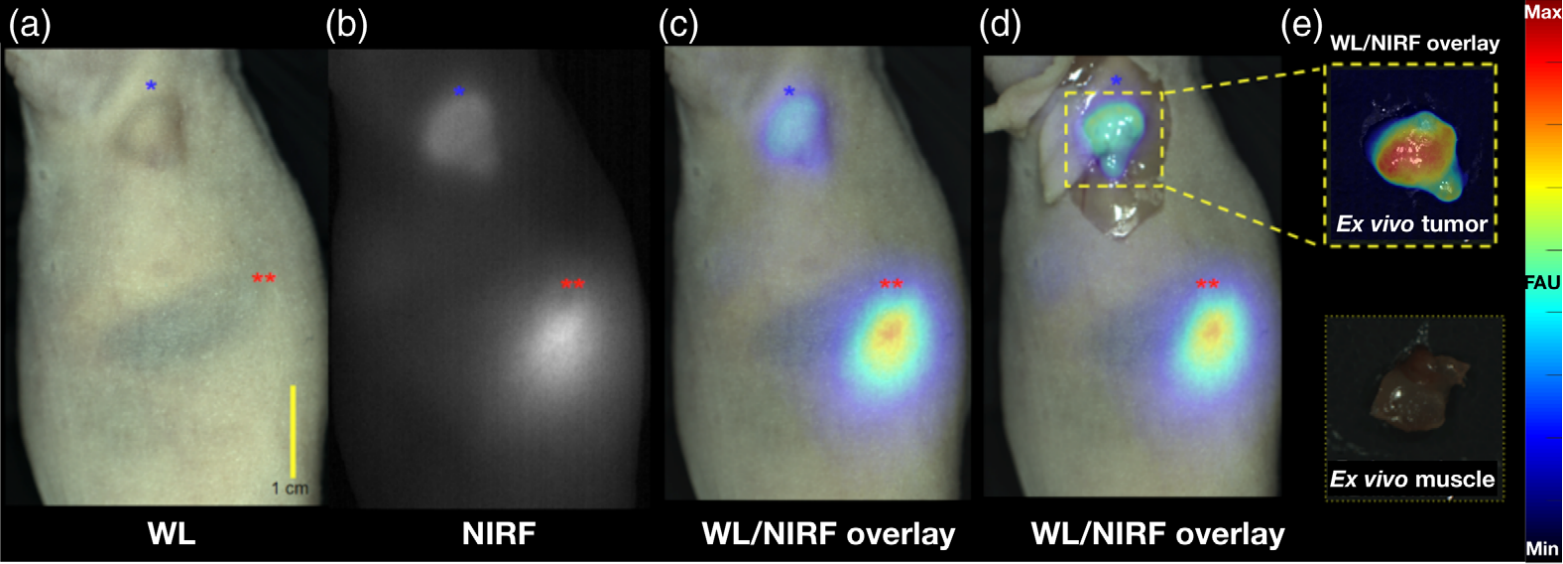
High *in vivo* contrast detection of fluorescently-labeled subcutaneous tumors under ambient light using simultaneous WL and NIRF image acquisition on the OnLume imager. Representative images of a HCT116-SSTR2 tumor imaged *in situ* 48 h after the injection of Ga67-MMC(IR800)-TOC under (a) WL, (b) NIRF, (c) WL with NIRF overlay, and (d) WL with NIRF overlay with skin retracted. (e) WL with NIRF overlay of excised tumor and muscle. The tumor is labeled by the blue star (*), and the kidney is labeled with two red stars (**). Fluorescence arbitrary units (FAU).

We evaluated the ability of the drug–device combination to provide *in vivo* tumor contrast and delineation under ambient light conditions that are representative of a standard-of-care surgical procedure. Based on our previous results, we selected 2 nmol as the injection dose and imaged at 48-h post-injection.^31^ Mice with HCT116-SSTR2 xenografts were imaged longitudinally using the OnLume imager and representative images are shown in Fig. 3 (cohort shown in Fig. 2 in the Supplementary Material). Tumors seen with WL capture [Fig. 3(a)] were also readily detected on the NIRF channel [Fig. 3(b)]. The combination of strong tumor-associated fluorescence and low background signal yielded high contrast tumor detection. As expected, notable kidney signal was present due to renal elimination of the drug. The overlay images [Figs. 3(c) and 3(d)] showed excellent co-localization of NIRF signal and tumor location, indicating accurate delineation of the subcutaneous xenograft with the drug–device combination. Tumor contrast was further improved upon *in situ* exposure of the tumor cavity [Fig. 3(d)] and evident on *ex vivo* imaging [Fig. 3(e)].

### 3.3 *Ex vivo* Contrast Analysis and Comparison of Image Quality Parameters for Optimal Dose Selection

**Fig. 4:**
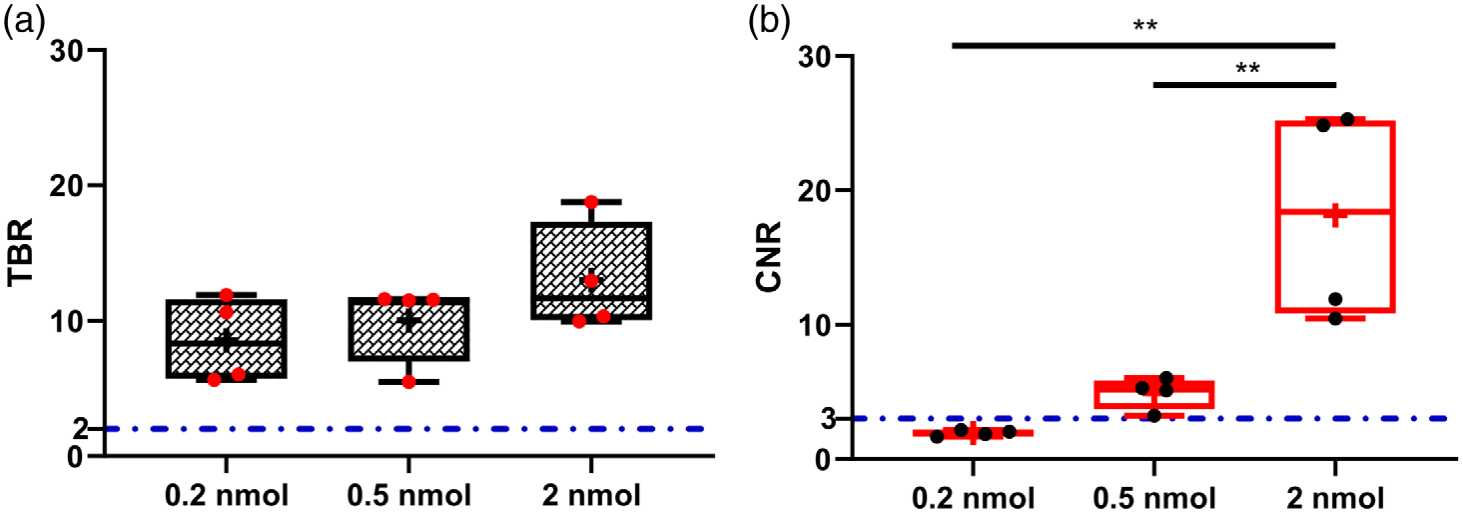
Tumor contrast as a function of Ga67-MMC(IR800)-TOC dose. Mice with HCT116-SSTR2 tumors were intravenously injected with 0.2, 0.5, or 2 nmol of drug. 24-h post-injection mice were sacrificed and tumor, muscle were excised and imaged with the OnLume imager to determine (a) TBR and (b) CNR. The dashed-dotted blue lines in (a) and (b) represent the commonly used threshold for TBR and the Rose criterion, respectively. The box plot extends from the 25th to 75th percentiles, the line in the middle indicates the median, the cross indicates the mean, and the whiskers extend from the minimum to the maximum value (n=4/dose). **, P<0.01.

Using resected HCT116-SSTR2 xenografts and muscle as background tissue, we evaluated *ex vivo* TBRs [Fig. 4(a)] and CNRs [Fig. 4(b)] produced by pairing the OnLume imager and MMC(IR800)-TOC under ambient light in a dose-finding study (2, 0.5, and 0.2 nmol at 24 h). Tissues were analyzed in parallel with the *In-Vivo* Xtreme small animal imaging device (Bruker) to provide comparative analysis with a standard preclinical imaging device. The range of TBRs across doses for the OnLume imager was 8.6±3.2 to 13±4.1 (P>0.05). We observed an increase in tumor CNR from 1.9±0.2 (0.2 nmol) to 18.1±8 (2 nmol) (P<0.01) that was dose-dependent. The TBRs and CNRs captured with the OnLume imager were in agreement with those from the Xtreme (Fig. 1 in the Supplementary Material), although the range of ratios across doses was narrower for the Xtreme [TBR range: 1.5±0.2 (0.2 nmol) to 5±0.9 (2 nmol), P<0.01]; CNR range: 0.7±0.3 (0.2 nmol) to 8±1.5 (2 nmol), P<0.01). We selected 2 nmol as the optimal imaging dose based on the superior CNR attained compared to the other doses.

### 3.4 *In vivo* Time-Course Visualization of Fluorescence Accumulation in Tumors and Clearance

**Fig. 5:**
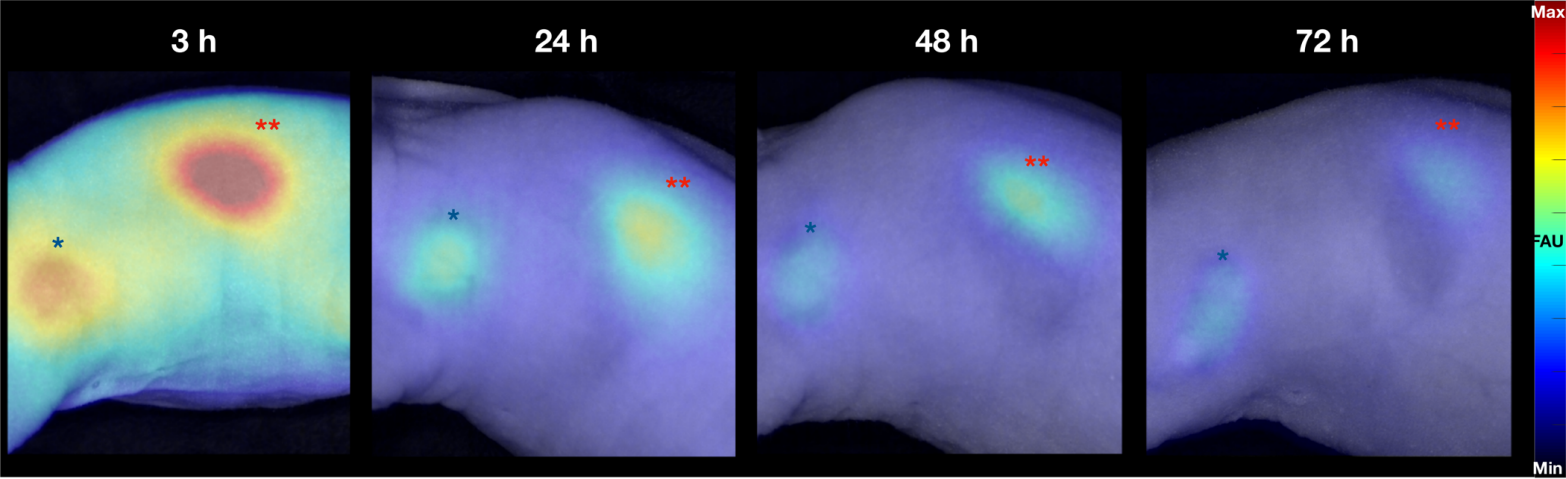
Qualitative assessment of the *in vivo* tumor contrast as a function of time. Mice with HCT116-SSTR2 tumors were intravenously injected with 2 nmol of Ga67-MMC(IR800)-TOC and imaged with the OnLume imager at 3, 24, 48, and 72 h post-injection (n=4/time point, cohort shown in Fig. 2 in the Supplementary Material). For these representative mice, NIRF images are all on the same relative scale and overlaid on the WL reflectance captured simultaneously under ambient light. The tumor is labeled by the blue star (*), and the kidney is labeled with two red stars (**). FAUs, fluorescence arbitrary units.

We previously showed that Ga68/67-MMC(IR800)-TOC binding to tumors can be visualized from 3- to 48-h post-injection using a small animal imaging device.^31^ As shown in Fig. 5, the detection window can be further expanded to 72-h post-injection. Images at 3 h show intense tumor fluorescence but also have notable signal in non-tumor sites. At 24 h, fluorescence in non-tumor tissues was reduced to background levels, leaving tumor and kidneys as the only detectable sites of NIRF signal. Despite a modest decrease in tumor fluorescence at 48 and 72 h, tumors were still clearly visible at delayed time points when using the same dynamic range and image processing parameters as the 3-h image.

### 3.5 *Ex vivo* Analysis of NET-Associated Tissues and Selection of Optimal Imaging Time Point

**Fig. 6:**
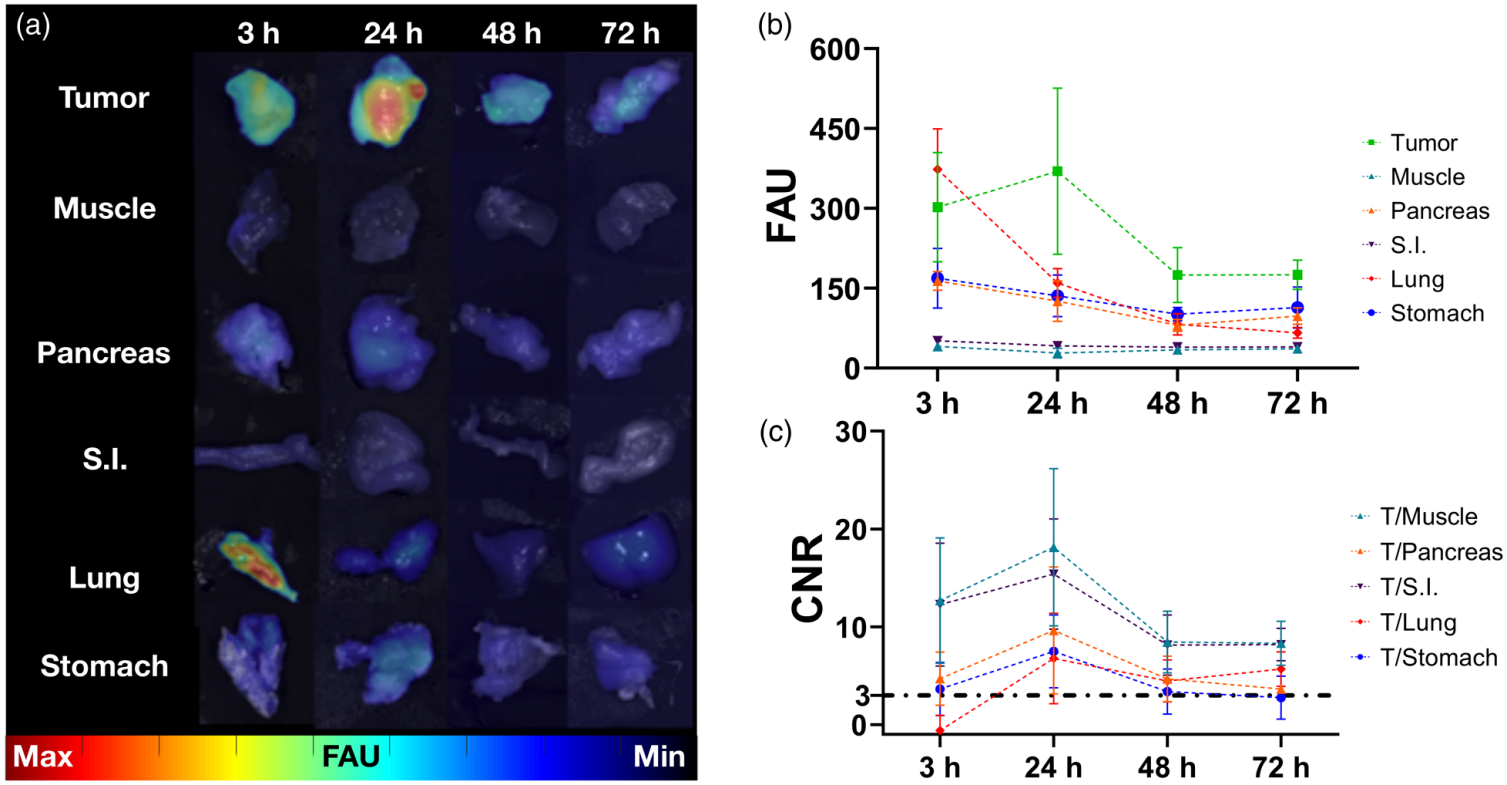
Qualitative and semi-quantitative *ex vivo* assessment of NET-associated tissues over time. (a) Longitudinal *ex vivo* imaging confirms preferential uptake in tumors. (b) Analysis of tissue fluorescence in FAU and (c) corresponding CNR at major sites of NET incidence. Data presented as mean±SD (n=4/time point). S.I., small intestine. The dashed-dotted black line in (c) represents the Rose criterion (CNR threshold).

Macroscopic evaluation by *ex vivo* imaging was in agreement with *in vivo* results and is summarized in Fig. 6. Imaging findings revealed notable tumor fluorescence at 3 h, which peaked at 24 h before gradually declining over the duration of the study. However, even at the 48 and 72 h, tumor fluorescence was still higher than healthy tissues with endogenous SSTR2 expression (pancreas, small intestine, lung, and stomach). This suggests that there is a large time window where GEP-NETs could potentially be detected intraoperatively by our drug–device combination [Fig. 6(a)]. High fluorescence was seen in the lungs at 3 h but was reduced to background levels by 24 h. Fluorescence in muscle was minimal throughout. Image analysis was performed to measure fluorescence readouts in each tissue and was in agreement with macroscopic results [Fig. 6(b) and Table 1 in the Supplementary Material]. Tumor signal was higher than all tissues (except for lung at 3 h) and remained constant up to 48 h (P>0.05). Conversely, fluorescence signal decreased over time in muscle (10.6% to 30.7% decrease), pancreas (23.3% to 51.1% decrease), small intestine (18.3% to 22.9% decrease), lung (57.3% to 82.3% decrease), and stomach (19.6% to 40.0% decrease). These results are in agreement with prior findings showing that retention of Ga68/67-MMC(IR800)-TOC in tissues is dependent on SSTR2 overexpression. Maximum CNRs were obtained at 24 h, where the 1.4 (muscle), 2.1 (pancreas), 1.3 (small intestine), 11.1 (lung), and 2.1 (stomach)-fold CNR increase from 3 to 24 h resulted in corresponding CNRs of 18.1±7 (muscle), 9.6±5.6 (pancreas), 15.4±4.9 (small intestine), 6.8±4 (lung), and 7.5±3.2 (stomach) [Fig. 6(c)].

### 3.6 Resection of SSTR2-Expressing Tumors Using the Drug–Device Combination

**Fig. 7:**
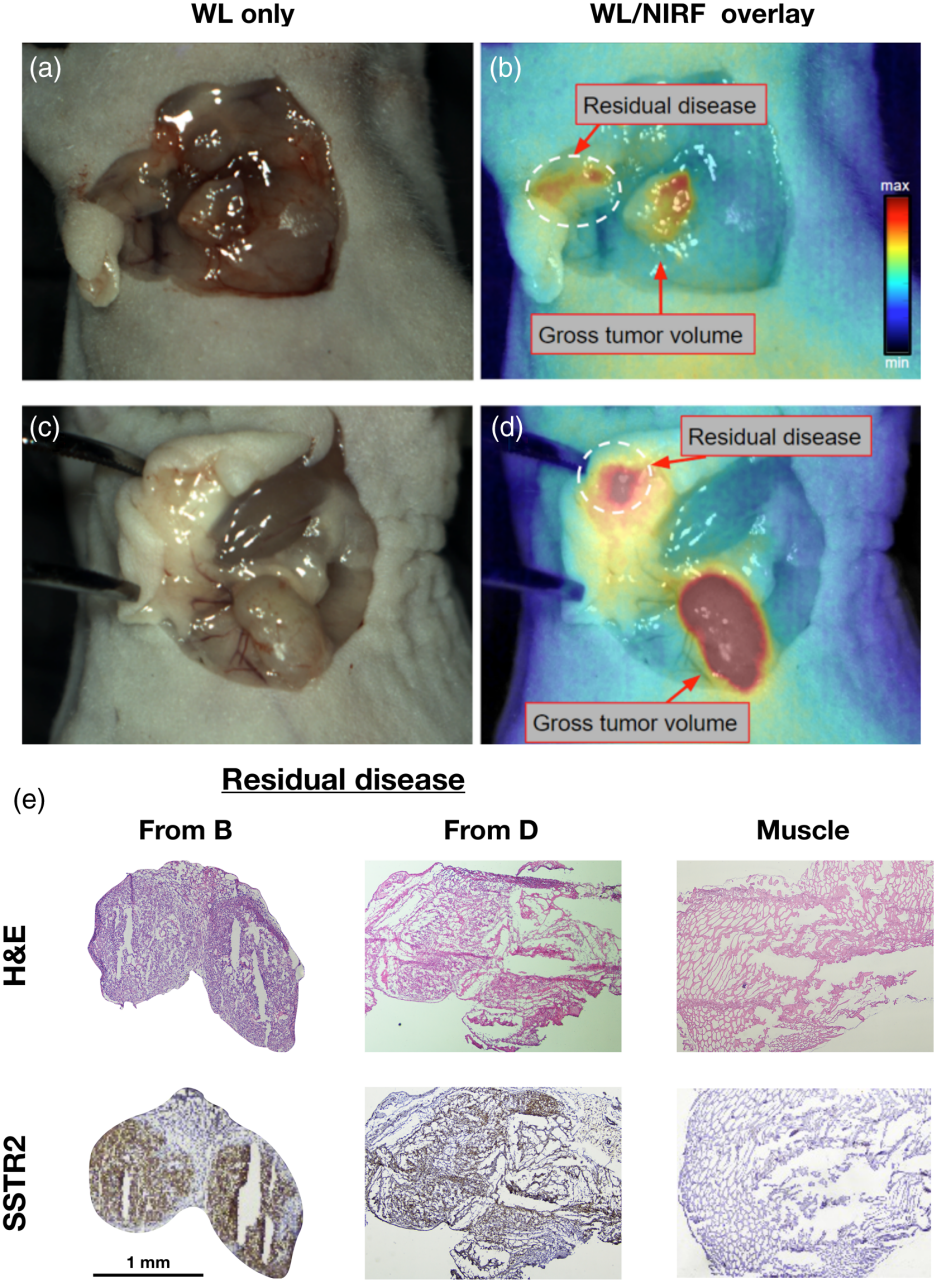
Real-time, intraoperative detection of residual disease using FGS under ambient light. (a) and (c) WL images of the wound bed after tumors were resected using visual inspection and palpation. (b) and (d) Corresponding NIRF images acquired with the OnLume imager revealed residual fluorescence that was not visible with the naked eye (dashed circle). (e) Histological analysis of these microlesions confirmed for cancer positivity (H&E) and SSTR2-expression (IHC). Muscle staining was performed as a negative control. Reproduced with permission from Ref. 34 (Video 1, MP4, 50 MB [URL: https://doi.org/10.1117/1.JBO.25.12.126002.1]) (Video 2, MP4, 47 MB [URL: https://doi.org/10.1117/1.JBO.25.12.126002.2]).

To evaluate the feasibility of using the drug–device combination for FGS, we performed resection of tumors in two mice 48 h after injection of Ga67-MMC(IR800)-TOC. Intraoperative guidance from the OnLume NIRF imaging system provided real-time, video-rate visualization of fluorescence overlay on WL reflectance without modification to sources of ambient light (Video 1 and Video 2). On whole-body imaging, high fluorescence signal was detected transdermally from the tumor and kidney in both mice. Throughout the tumor resection procedure, fluorescence was well visualized and the associated contrast allowed clear delineation from surrounding tissues. After tumor removal, the wound bed was surveyed with the OnLume imager for any remaining fluorescence. FGS enabled *in situ* detection of residual fluorescence in tissues that were not initially identified as lesions during visual and tactile inspection by the surgeon. The residual cancerous tissues were not readily recognized as tumor because they lacked the fibrotic characteristics of the resected tumors and instead had the consistency of adipose tissue. Immunohistochemical analysis of suspicious lesions confirmed the presence of SSTR2-positive disease and demonstrated the high specificity of Ga67-MMC(IR800)-TOC for tumor-specific targeting while simultaneously showing the high detection sensitivity and spatial resolution of the device for detecting residual fluorescence in lesions that measured <2  mm in size (Fig. 7).

### 3.7 Biodistribution and Relationship between the Underlying Drug Distribution and Fluorescence Intensity

**Fig. 8:**
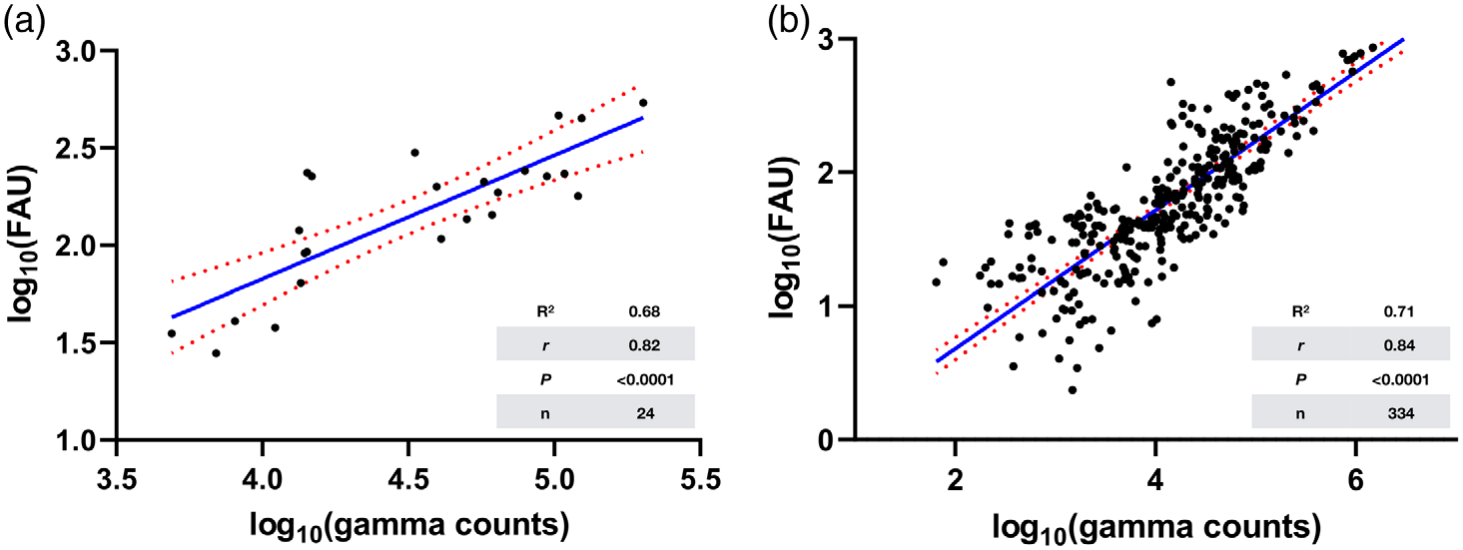
Relationship between the underlying drug distribution and the fluorescence acquired by the OnLume imager. Fitting of log-transformed linear regression models using gamma counts (drug only) and fluorescence arbitrary units (FAU; drug–device) in (a) tumors (n=24) and (b) all tissues (n=334) as independent and dependent variables, respectively.

Given the importance of developing new methods for quality control in fluorescence-based imaging techniques, we used the radiolabeling properties of Ga67-MMC(IR800)-TOC to quantify absolute drug concentration in excised tissues to obtain % injected dose per gram of tissue (%ID/g), which is a standardized value in nuclear medicine. As shown in Tables 1 to 4 in Supplementary Material, raw fluorescence arbitrary units (FAUs) acquired by the OnLume imager had nearly identical trends to %ID/g values. We then implemented a log-transformed linear regression model to evaluate the relationship between the fluorescence readouts and the quantitative nuclear reporter of the drug. This analysis showed that as the amount of gamma counts in tumors increase, FAUs also increase ($R^{2}=0.68$, r=0.82, n=24, P<0.0001) [Fig. 8(a)]. Remarkably, when all tissues (n=334 from 24 mice at all doses (0.2, 0.5, and 2 nmol) and time points (3, 24, 48, and 72 h)) are included in the model, the linearity and correlation between fluorescence and radioactivity was maintained ($R^{2}=0.71$; r=0.84; P<0.0001) [Fig. 8(b)].

## 4 Discussion

It has been nearly 10 years since the seminal study that demonstrated first-in-human application of a tumor-specific contrast agent for FGS,^36^ and the ensuing development of drugs for a wide range of cancers has been substantial.^37^ However, the translation of preclinical technologies into clinical practice has been slow due in part to limited validation strategies for drugs, devices and their combined use.^38^ Our approach for developing a targeted FGS drug builds upon the clinical use of radiolabeled somatostatin analogs, which have revolutionized the evaluation and management of NETs.^39^ Validation of a fluorescent somatostatin analog could bring similar clinical benefit into the operating room. This approach not only enables the use of Food and Drug Administration-approved radiotracers for comparative analysis of binding and pharmacokinetic properties, but also lowers the risks associated with the development of a tumor-specific drug for FGS.^40^ Benchmarking with an *in vivo* gold standard is unique to SSTR2-targeting strategies and provides insight that would otherwise not be available during drug development. However, to provide meaningful intraoperative information to surgeons in real-time, key image acquisition elements must be present as discussed below.

The quality of an intraoperative image depends largely on signal contrast between tumor and adjacent non-tumor tissue. Low drug uptake, even under ideal minimal background uptake conditions, may be below the detection sensitivity threshold of FGS imagers and may lead to poor image quality. Thus, an FGS agent must be given at a dose that (i) robustly accumulates in tumors to form a “fluorescent depot” and (ii) generates high image contrast. To evaluate tumor contrast as a function of dose, we used TBR and CNR as image quality parameters (Fig. 4). TBR is a commonly used measurement that only describes how much more signal is captured in one region-of-interest (ROI) compared to another. CNR provides information regarding how well a fluorescent ROI (i.e., tumor) can be discerned from the background (i.e., tumor-associated healthy tissue and image noise) and thus, measures the quality of the visual contrast.^41^ It is important to note that thresholds and image quality parameters to be used during preclinical drug–device development are not well established.^42^ Interestingly, we found that although TBRs exceeded the recommended threshold of 2^42^ for all doses, there was no difference across dose groups (P>0.05) and did not provide a meaningful rationale for selecting one dose over another. TBRs gained with our drug–device combination (range: 8.6±3.2 to 13±4.1) compared favorably to previously reported values using fluorescently-labeled small molecules and peptides. For instance, an antagonist of gastrin-releasing peptide receptor^43^ and prostate-specific membrane antigen conjugate^44^ for FGS in prostate cancer were shown to provide TBRs (muscle) ranging from ∼7.5 to 25. Conversely, CNRs were >3 in two of three doses evaluated (0.5 nmol: 4.9±1.2; 2 nmol: 18.1±8.0), which is critical for visually distinguishing image features with certainty (Rose criterion).^45^ 2-nmol yielded the largest CNR improvement over the Rose criterion and was the rationale we used for selecting it as the optimal dose for subsequent testing.

The invariable pharmacokinetic properties of the drug are largely responsible for the signal seen in non-tumor tissues and play an important role in determining the imaging time point used in FGS. This relationship has high clinical relevance as it could ultimately provide imaging parameters that enhance true positive detection rates while decreasing false positives.^9^ Accordingly, a key clinical endpoint of phase I studies with FGS agents is to identify the imaging time point with the highest image contrast. Since physiologic expression of SSTR2 is limited to only a few normal tissues (e.g., pancreas, adrenals, lungs, small intestine, and spleen) where expression is lower than in typical NETs, the persistent Ga67-MMC-(IR800)-TOC signal in tumors up to 72-h post-injection (Fig. 5) suggests robust clinical imaging potential. Consistent with our prior reports,^31^ rapid elimination of Ga67-MMC-(IR800)-TOC from normal tissues produced *ex vivo* CNRs >3 in nearly all relevant tissues (with the exception of lung) at 3 h. Continued clearance at 24-h produced maximal CNR values that well exceeded the Rose criterion and ranged from 6.8±4 (lung) to 18.1±7 (muscle) (Fig. 6). Most notably, this includes GEP-NET sites that commonly present with localized or metastatic lesions, such as lung and the GEP endocrine system [9.6±5.6 (pancreas), 15.4±4.9 (small intestine), and 7.5±3.2 (stomach)]. Should logistical considerations or the complexity of surgical procedures become an issue, the ability of the agent to produce CNR values >3 at 48 and 72 h could potentially provide flexibility in the design and conduct of SSTR2-targeted FGS.

The tumors in this study were <1  cm in diameter, which are common in surgical cases of GEP-NETs for which we need reliable tumor detection technology, and yet were well visualized by our drug–device combination. Moreover, the intraoperative detection of post-resection fluorescence determined to be residual disease (<2  mm) (Fig. 7) shows that the OnLume NIRF imaging system can robustly localize fluorescently-labeled, SSTR2-expressing tumors that are comparable in size to clinical scenarios. Thus, there is a high likelihood that NETs can be distinguished from other tissues and structures in the wound bed with Ga67-MMC(IR800)-TOC. This would represent a major advance in oncologic surgery and reduce the dependence on time-consuming histopathology feedback to determine whether R0 resection has been achieved. Further benefits would be seen in healthcare costs, which, in turn, would be lower as operating time is reduced.

Even if an FGS agent possesses the above-mentioned characteristics, clinical utility will only be realized if the imaging device has the necessary opto-mechanical specifications (i.e., sensitivity, filter sets, and form factor) to accurately assess its performance. Since clinical FGS systems are optimized for ICG (peaks of excitation/emission: 780/830  nm), detection of different NIRF dyes (e.g., IRDye800: Ex/Em 774/789  nm) or dye conjugates may be less efficient and underestimate agent performance. As a result, the absence of fluorescence in a previously defined tumor region may in fact be a false negative that inaccurately reports undetectable fluorescence (e.g., due to poor detection sensitivity) as a lack of cancerous tissue. Therefore, the present study examined the combined use of the benchtop OnLume NIRF imaging system with Ga67-MMC(IR800)-TOC in an intraoperative setting that is not possible with existing preclinical small animal imaging devices (Video 1 and Video 2). The OnLume system was designed as a preclinical fluorescence imaging system that mimics intraoperative FGS with ambient light capability and the ability to acquire snapshots and videos in real-time (e.g., no perceptible latency and video-rate display). Although the system was initially designed for compatibility with ICG, its modular optical components were easily modified to optimally image Ga67-MMC(IR800)-TOC (Fig. 2).

The objective of FGS is to visualize the underlying distribution and concentration of a fluorescent drug in real-time. If the efficacy of FGS solely depended on drug distribution and concentration, the relationship between fluorescence strength and drug concentration should be linear.^46^ However, fluorescence imaging does not provide a true indication of drug distribution, but rather a visual composite of many factors that are associated with drug performance *in vivo* and device characteristics.^47^ Given that our FGS drug development strategy is built upon the design of a radiopharmaceutical, we are equipped with the ability to dual-label our drug with radionuclides that overcome the detection-depth and device-acquisition limits of NIRF imaging.^46^ We initially used the radiolabeling utility of the MMC to produce Ga67-MMC(IR800)-TOC for (i) quantification of absolute drug distribution as %ID/g (Tables 3 and 4 in the Supplementary Material) and (ii) cross-validation of trends from fluorescent-based detection methods (Tables 1 and 2 in the Supplementary Material). We then assessed the variability of the FAUs (dependent/response variable; imager) as a function of gamma counts (independent/predictor variable; drug) (Fig. 8) and found the relationship to be linear, potentially due to the combination of small tumor and tissue size (low scattering) and the high sensitivity of the device. Given that fluorescent- and radioactive-based detection methods have significantly different dynamic ranges, we log-transformed both scales to visually appreciate the relationship. Results showed a strong agreement (tumors only: $R^{2}=0.68$, r=0.82, P<0.0001, n=24; all tissues: $R^{2}=0.71$, r=0.84, P<0.0001, n=334) between absolute drug distribution and imaging under ambient light. To the best of our knowledge, this cross-validation methodology had not been reported previously as part of FGS drug–device development. Given the lack of established quantitative methods for evaluating drug–device combinations, we envision the use of dual labeling as an additional preclinical tool to guide and optimize FGS development strategies. Furthermore, the most significant impact of dual labeling may occur within drug optimization as novel NIRF dyes with superior pharmacokinetic and optical properties continue to emerge.^48^
