# Supplementary material for: Development of a drug–device combination for fluorescence-guided surgery in neuroendocrine tumors
Source: J Biomed Opt. 2020 Dec 9;25(12):126002. doi: 10.1117/1.JBO.25.12.126002 (PMC7725236; doi:10.1117/1.JBO.25.12.126002)
Supplement: Supplementary file 1 [file JBO_025_126002_SD001.pdf]

Supplementary information

Development of a drug-device combination for fluorescence-guided surgery in neuroendocrine tumors

Servando Hernandez Vargas[1,*], Christie Lin[2,*], Julie Voss[1], Sukhen C. Ghosh[1], Daniel M. Halperin[3], Solmaz AghaAmiri[1], Hop S. Tran Cao[4], Naruhiko Ikoma[4], Adam J. Uselmann[2], Ali Azhdarinia[1]

[1]The Brown Foundation Institute of Molecular Medicine, McGovern Medical School, The University of Texas Health Science Center at Houston (Houston, TX, USA)

[2]OnLume, Inc. (Madison, WI, USA)

[3]Department of Gastrointestinal Medical Oncology, Division of Cancer Medicine, The University of Texas MD Anderson Cancer Center (Houston, TX, USA)

[4]Department of Surgical Oncology, Division of Surgery, The University of Texas MD Anderson Cancer Center (Houston, TX, USA)

*Co-first author

Corresponding author: Ali Azhdarinia, The University of Texas Health Science Center at Houston, 1881 East Rd., 3SCR6.4680, Houston, TX 77054. Phone: 713- 500-3577; Fax: 713-500-0319; E-mail: ali.azhdarinia@uth.tmc.edu

Running title: Drug-device combination for FGS in NETs

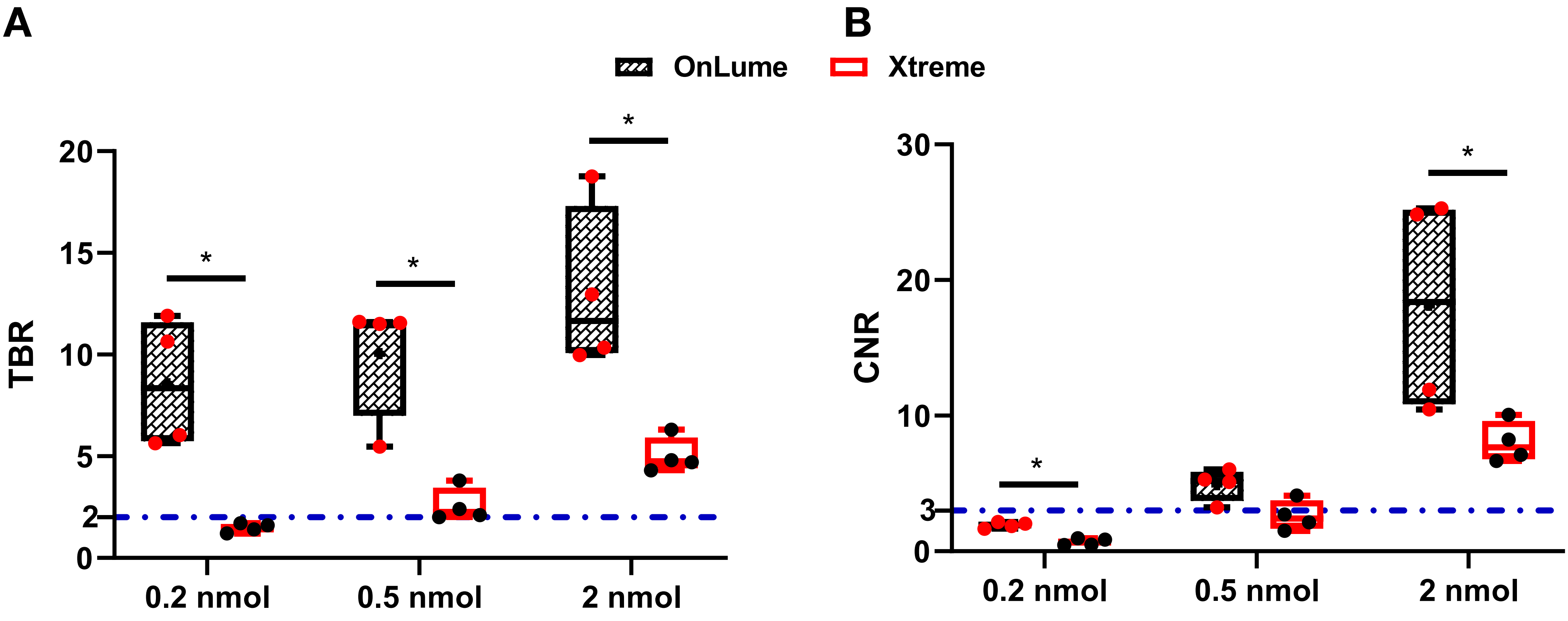

Supplementary Figure 1. Comparison of tumor contrast on the OnLume imager and In-Vivo Xtreme (preclinical small animal imaging device) as a function of ^{67}Ga-MMC(IR800)-TOC dose. Mice with HCT116-SSTR2 tumors were intravenously injected with 0.2, 0.5 or 2 nmol of drug. 24 hours post-injection mice were sacrificed and tumor, muscle were excised and imaged with the OnLume imager and In-Vivo Xtreme (Bruker) to determine (A) TBR and (B) CNR. The dashed-dotted blue lines in (A) and (B) represent the commonly used treshold for TBR and the Rose criterion, respectively. The box plot extends from the 25th to 75th percentiles, the line in the middle indicates the median, the cross indicates the mean, and the whiskers extend from the minimum to the maximum value (n = 4/dose). *, $P<0.05$.

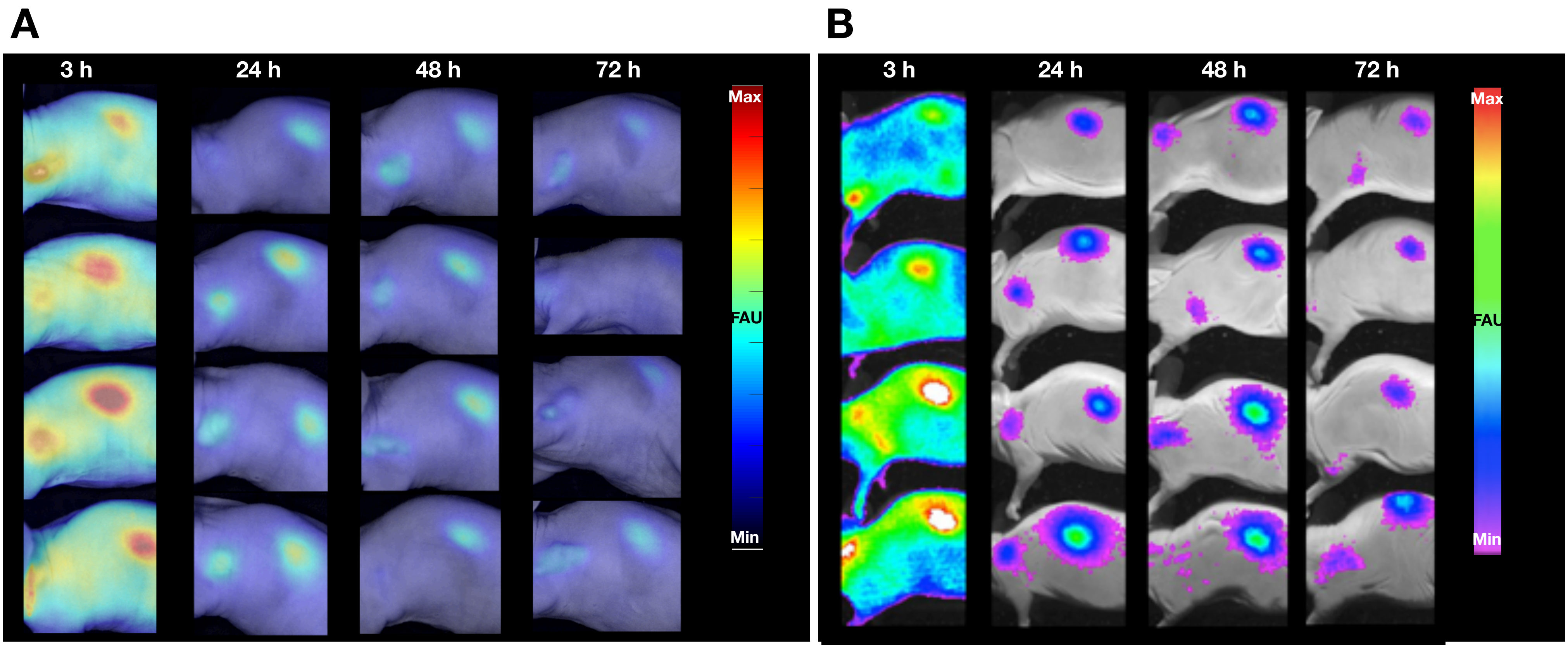

Supplementary Figure 2. *In vivo* time-course fluorescence imaging of mouse cohorts injected with optimal dose. Mice with HCT116-SSTR2 tumors were intravenously injected with 2 nmol of drug and imaged longitudinally over time using the (A) OnLume imager and (B) In-Vivo Xtreme (n = 4/time point).

Supplementary Table 1. Fluorescence arbitrary units (FAUs) for 2 nmol of ^{67}Ga-MMC(IR800)-TOC in HCT116-SSTR2 xenografts at 3, 24, 48 and 72 h post-injection (n = 4/time point). S.I., small intestine.

	**3 h**	**24 h**	**48 h**	**72 h**
**Tumor**	302.3 ± 102.7	369.7 ± 156.1	174.9 ± 51.8	175.4 ± 27.7
**Muscle**	40.8 ± 4.4	28.3 ± 8.9	34.5 ± 5.2	36.5 ± 4.7
**Pancreas**	163.9 ± 17.9	125.7 ± 38.1	80.2 ± 11.2	97.7 ± 15.4
**S. I.**	51.0 ± 8.7	41.7 ± 6.6	39.3 ± 1.5	39.6 ± 5.7
**Lung**	373.2 ± 75.8	159.5 ± 27.5	82.2 ± 20.2	66.1 ± 9.9
**Liver**	329.6 ± 30.6	266.4 ± 23.0	196.7 ± 29.3	158.9 ± 37.2
**Spleen**	103.8 ± 18.0	55.8 ± 11.4	54.4 ± 6.2	46.7 ± 5.9
**Stomach**	169.0 ± 56.5	135.9 ± 38.9	101.4 ± 12.8	113.5 ± 38.9
**Bone**	213.9 ± 45.9	103.8 ± 25.8	75.4 ± 9.1	64.9 ± 6.7
**Heart**	116.6 ± 56.7	53.5 ± 19.6	48.8 ± 10.0	46.7 ± 8.8
**Adrenal**	57.4 ± 7.0	40.5 ± 10.6	46.8 ± 6.4	46.7 ± 1.4
**Kidney**	1641.4 ± 105.9	1704.7 ± 178.5	809.8 ± 186.5	729.3 ± 36.2
**Brain**	27.0 ± 9.5	27.0 ± 8.3	38.7 ± 3.4	37.7 ± 3.3
**Skin**	429.6 ± 85.0	203.2 ± 50.9	103.6 ± 14.7	108.5 ± 14.5

Supplementary Table 2. Fluorescence arbitrary units (FAUs) for 0.2, 0.5 and 2 nmol of ^{67}Ga-MMC(IR800)-TOC in HCT116-SSTR2 xenografts at 24 h post-injection (n = 4/time point). S.I., small intestine.

	0.2 nmol	0.5 nmol	2 nmol
**Tumor**	35.5 ± 2.8	88.9 ± 18.3	369.7 ± 156.1
**Muscle**	4.3 ± 5.5	9.2 ± 1.7	28.3 ± 8.9
**Pancreas**	16.0 ± 1.6	36.1 ± 9.3	125.7 ± 38.1
**S. I.**	10.0 ± 4.8	14.2 ± 2.4	41.7 ± 6.6
**Lung**	12.3 ± 6.5	37.0 ± 9.3	159.5 ± 27.5
**Liver**	31.2 ± 9.2	77.9 ± 21.0	266.4 ± 23.0
**Spleen**	8.9 ± 5.0	13.4 ± 6.9	55.8 ± 11.4
**Stomach**	18.0 ± 6. 3	37.6 ± 8.7	135.9 ± 38.9
**Bone**	18. 3 ± 8.6	36.2 ± 3.8	103.8 ± 25.8
**Heart**	8.1 ± 6.8	19.5 ± 2.7	53.5 ± 19.6
**Adrenal**	15.1 ± 4.8	16.5 ± 2.8	40.5 ± 10.6
**Kidney**	126.1 ± 23.7	412.00 ± 52.9	1704.7 ± 178.5
**Brain**	18.1 ± 2.7	18.7 ± 3.0	27.0 ± 8.3
**Skin**	32.3 ± 1.3	65.1 ± 5. 3	369.7 ± 156.1

Supplementary Table 3. Biodistribution results (%ID/g) for 0.2, 0.5 and 2 nmol of ^{67}Ga-MMC(IR800)-TOC in HCT116-SSTR2 xenografts at 24 h post-injection (n = 4/time point). S.I., small intestine.

	0.2 nmol	0.5 nmol	2 nmol
**Tumor**	3.75 ± 0.98	6.48 ± 1.87	9.69 ± 2.50
**Muscle**	0.28 ± 0.27	0.31 ± 0.04	0.62 ± 0.27
**Pancreas**	1.24 ± 0.14	2.20 ± 0.85	2.98 ± 0.87
**S. I.**	0.82 ± 0.60	1.01 ± 0.49	2.89 ± 2.24
**Lung**	1.71 ± 0.57	3.03 ± 0.64	6.89 ± 0.94
**Liver**	2.96 ± 0.87	6.11 ± 0.69	8.14 ± 1.26
**Spleen**	1.20 ± 0.16	2.53 ± 1.36	3.66 ± 0.58
**Stomach**	1.81 ± 0.83	3.55 ± 1.78	5.62 ± 2.14
**Bone**	0.73 ± 0.14	1.40 ± 0.31	1.96 ± 0.42
**Heart**	0.34 ± 0.08	0.69 ± 0.10	1.04 ± 0.18
**Adrenal**	0.92 ± 0.20	3.54 ± 3.16	2.50 ± 0.90
**Kidney**	17.17 ± 3.53	28.00 ± 4.08	46.75 ± 5.28
**Brain**	0.02 ± 0.0	0.03 ± 0.01	0.12 ± 0.14
**Blood**	0.17 ± 0.04	0.27 ± 0.04	0.44 ± 0.12
**Skin**	1.10 ± 0.26	1.98 ± 0.13	2.45 ± 0.17

Supplementary Table 4. Biodistribution results (%ID/g) for 2 nmol of ^{67}Ga-MMC(IR800)-TOC in HCT116-SSTR2 xenografts at 3, 24, 48 and 72 h post-injection (n = 4/time point). S.I., small intestine.

	**3 h**	**24 h**	**48 h**	**72 h**
**Tumor**	6.26 ± 2.28	9.69 ± 2.50	8.40 ± 1.24	4.28 ± 0.84
**Muscle**	1.05 ± 0.17	0.62 ± 0.27	0.41 ± 0.33	0.22 ± 0.04
**Pancreas**	2.50 ± 0.66	2.98 ± 0.87	1.72 ± 0.52	1.84 ± 0.45
**S. I.**	1.92 ± 0.82	2.89 ± 2.24	1.19 ± 0.44	0.51 ± 0.06
**Lung**	14.99 ± 0.35	6.89 ± 0.94	2.27 ± 0.30	1.21 ± 0.30
**Liver**	7.03 ± 0.91	8.14 ± 1.26	5.52 ± 0.80	3.74 ± 0.49
**Spleen**	5.57 ± 0.99	3.66 ± 0.58	2.05 ± 0.35	1.56 ± 0.26
**Stomach**	5.38 ± 1.21	5.62 ± 2.14	2.28 ± 0.49	1.53 ± 0.79
**Bone**	3.24 ± 0.80	1.96 ± 0.42	1.21 ± 0.24	0.98 ± 0.15
**Heart**	1.90 ± 0.40	1.04 ± 0.18	0.52 ± 0.13	0.42 ± 0.09
**Adrenal**	2.78 ± 0.65	2.50 ± 0.90	1.51 ± 1.26	1.08 ± 0.22
**Kidney**	42.82 ± 3.13	46.75 ± 5.28	29.20 ± 4.14	21.99 ± 1.54
**Brain**	0.05 ± 0.01	0.12 ± 0.14	0.02 ± 0.01	0.02 ± 0.01
**Blood**	1.04 ± 0.50	0.44 ± 0.12	0.18 ± 0.04	0.13 ± 0.03
**Skin**	4.92 ± 1.90	2.45 ± 0.17	1.36 ± 0.58	1.19 ± 0.20
